# Biological Effect of Leaf Aqueous Extract of *Caesalpinia pyramidalis* in Goats Naturally Infected with Gastrointestinal Nematodes

**DOI:** 10.1155/2012/510391

**Published:** 2012-04-04

**Authors:** Roberto Robson Borges-dos-Santos, Jorge A. López, Luciano C. Santos, Farouk Zacharias, Jorge Maurício David, Juceni Pereira David, Fernanda Washington de Mendonça Lima

**Affiliations:** ^1^Escola de Medicina Veterinária, Universidade Federal da Bahia, 40170-000 Salvador, BA, Brazil; ^2^Programa de Pós-Graduação em Biotecnologia Industrial, Instituto de Tecnologia e Pesquisa, Universidade Tiradentes, 49032-490 Aracaju, SE, Brazil; ^3^Centro de Pesquisa Gonçalo Moniz-FIOCRUZ, 40296-710 Salvador, BA, Brazil; ^4^Empresa Baiana de Desenvolvimento Agrícola, 41635-150 Salvador, BA, Brazil; ^5^Instituto de Química, Universidade Federal da Bahia, 40170-290 Salvador, BA, Brazil; ^6^Faculdade de Farmácia, Universidade Federal da Bahia, 41.170-280, Salvador, BA, Brazil

## Abstract

Forty-eight goats naturally infected with gastrointestinal nematodes were randomly divided into four groups (*n* = 12): negative control (G1) (untreated), positive control (G2) (treated with doramectin, 1 mL/50 Kg b.w.), and G3 and G4 treated with 2.5 and 5 mg/Kg b.w. of a leaf aqueous extract of *Caesalpinia pyramidalis* (CP). Fecal and blood samples were regularly collected for the evaluation of fecal egg count (FEC), hematological and immunological parameters to assess the anthelmintic activity. In treated animals with CP, there was noted a significant reduction of 54.6 and 71.2% in the mean FEC (*P* < 0.05). An increase in IgA levels was observed in G3 and G4 (*P* < 0.05), during the experimental period, suggesting that it was stimulated by the extract administration. In conclusion, the results showed that CP provoked a protective response in infected animals treated with them. This response could be partly explained by the CP chemical composition.

## 1. Introduction

Goat herding is an attractive source of income for farmers in northeastern Brazil. However, gastrointestinal nematodes (GINs) remain one of the greatest limiting factors to successful and sustainable livestock production worldwide [1]. Therefore, economic losses caused by GIN are related to decreased production, treatment costs, and even animal death in small ruminant production [2]. In semiarid Brazilian region, haemonchosis causes severe economic losses in livestock production (e.g., weight loss, stunted growth, and death) [3]. Its control based on the use of commercial anthelmintics is no longer considered sustainable due to an increased prevalence of GIN resistance as well as chemical residue and toxicity problems [2]. For these various reasons, interest in the plant screening for their anthelmintic activity remains of great scientific interest despite extensive use of synthetic chemicals in modern clinical practices all over the world as an alternative source of anthelmintics [4–6]. There are several species of plants used by traditional communities in the Brazilian semiarid.

The genus *Caesalpinia *(Fabaceae), composed of tropical or subtropical trees and shrubs, contains more than 150 species, spread throughout the world [7]. *Caesalpinia pyramidalis* Tul. (CP), known regionally as “catingueira” and “pau-de-rato”, is used as fodder in animal feed [8] and in the traditional medicine [9, 10]. Some compounds from *C. pyramidalis* leaves have previously been reported, such 4-*O*-*β*-glucopyranosyloxy-Z-7-hydroxycinnamic acid, lupeol, aghatisflavone, and other phenolics [10–13]. 

This work aims at evaluating the anthelmintic effect of CP extract *in vivo* against GIN natural infected goats in order to confirm the benefits.

## 2. Material and Methods

### 2.1. Plant Material and Aqueous Extract Preparation

*C. pyramidalis* leaves were collected in the caatinga region from Bahia, Brazil. A voucher herbarium specimen (number 0240291), classified systematically by Professor Letícia Scardino (Instituto de Biologia, UFBA), was deposited at Herbarium Alexandre Leal Costa (UFBA).

Leaves were air dried in an oven at 40°C. The powdered material (200 g) was extracted with boiling water for 20 min. The extract was filtered and lyophilized for bioassay analysis.

### 2.2. *Haemonchus contortus* Antigen Preparation

*H. contortus* adults of either sex, harvested from abomasums of slaughtered goats, were washed in PBS (pH 7.2) to prepare a crude extract by disrupting parasites in PBS pH 7.2 at 4°C by 30 s periods of ultrasonic treatment. After centrifugation (1200 g, 15 min, 4°C), the supernatant was either used immediately or stored at −20°C. The extract was used as the test antigen in ELISA.

### 2.3. Animals

The study was carried out with a total of 48 mixed bred goats selected from herded animals maintained at the Experimental Station of Bahia Company for Agro-Livestock Development (EBDA) in Jaguarari, Bahia, Brazil. Goats were traditional grazing under extensive management practices in the Brazilian semiarid region and had naturally acquired gastrointestinal nematode infection, being confirmed prior the experiment beginning by faeces egg count (FEC) (average values before starting the trial G: 483,33 ± 440,73; G2: 375,00 ± 344,11; G3: 591,67 ± 802,79; G4: 491,67 ± 454,19). Animals were randomly divided into four equal groups (*n* = 12). The first group (G1) served as the negative control and received no treatment while second group (G2) was the positive control treated with doramectin (1 mL/50 Kg b.w., Dectomax, Pfizer). The third and fourth groups (G3 and G4) were drenched with CP extract (2.5 and 5.0 mg/Kg b.w., resp.). The anthelmintic doramectin and the extract were administered through three consecutive days from the time zero.

Goats were kept under free management and grazed on the harvested crops, remnants of the fodder and vegetables, herbs, weeds, and shrubs along the banks of water channels throughout the experiment and monitored by examination of blood and faecal samples for immunological and parasitological parameters at 30 and 60 days after treatment. Animals were weighed (average weight before starting the trial G1: 14,70 ± 2,86; G2: 13,45 3,09; G3: 15,10 ± 2,96; G4: 17,0 ± 2,09) at stage zero and five days before the collection intervals to minimise the handling stress effects. The monitoring was carried out between March and May, coinciding with the end of the dry season and beginning of the rainy period. The experiment was conducted in accordance with the guidelines for care and use of experimental animals of the Brazilian College of Experimental Animal (COBEA).

### 2.4. FEC and Blood Sampling

Faecal and blood samples were recovered at day 0 and at 30 and 60 days after treatment. For FEC determination, faeces (2 g) were collected directly from the rectum and processed using a modified McMaster technique and expressed as eggs per gram [14]. 

Blood with and without anticoagulant (Na_2_EDTA; 1 mg/mL) was individually collected by jugular venipuncture. Blood collected with anticoagulant was used for monitoring red and white blood cell counts, expressed in number of cell/mm^3^. The packed cell volume (PCV) was measured by the microhaematocrit method. Sera were separated from blood samples without anticoagulant and stored at −20°C until required.

### 2.5. Antibody Responses

An ELISA was used to detect total IgG and IgA and specific anti-*Haemonchus* IgG in serum. For anti-*Haemonchus* serum IgG levels, plates were coated with an antigen solution (40 *μ*g/well in 50 mM carbonate buffer pH 9.6) by incubating at 4°C for 20 h. Afterwards, 100 *μ*L serum samples (1 : 200 diluted in PBS/0.05% Tween/0.25% defatty powered milk) were added in duplicate and incubated (1 h/37°C). After washing, 100 *μ*L of optimally diluted anti-goat HRP-conjugated IgG (Bethyl Inc. Montgomery, Texas, USA) was added and incubated (1 h/37°C). After washing, 100 *μ*L of *o*-phenylenediamine (6 mg in 15 mL of 0.1 M citrate phosphate buffer pH 5.0) and 10 *μ*L of H_2_O_2_ were added to wells. After 20 min of incubation, the reaction was stopped with 50 *μ*L of 0.5 M H_2_SO_4_. Optical Density (OD) at 450 nm was measured in an ELISA plate reader (Stat Fax, USA).

For total IgA and IgG dosages, the same protocol was employed, using a commercial kit (Bethyl Inc. Montgomery, TX, USA). Dosage standard curves were obtained following the protocols provided by the manufacturer.

### 2.6. Statistical Analysis

Results were expressed as means ± standard deviation (SD), using Kruskal-Wallis nonparametric (ANOVA on ranks) tests and multiple comparison tests, such as Dunn. *P* < 0.05 was considered statistically significant. All of the analyses were performed using the statistical software Graph Pad (San Diego, USA).

## 3. Results

### 3.1. Parasitological and Zootechnical Measures

FEC determination was carried out in four regular intervals, initially, two weeks before assay, with the aim at tracing and randomly distributing the animals into four groups at day zero, prior treatment (extract and doramectin), and then at days 30 and 60 after treatment in all groups.

**Figure 1:**
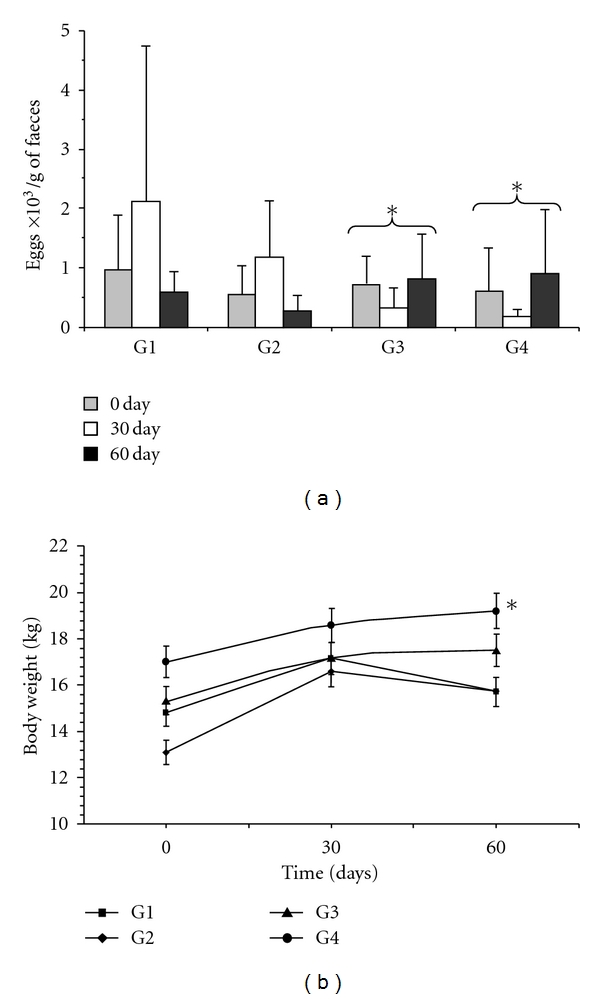
Mean number of nematode egg per gram of faeces in naturally infected goats (a) and mean live weight (b) along the experimental period. Values are means ± standard deviation. An asterisk mark represents significant differences; *P* < 0.05. G1: negative control (untreated); G2: positive control (treated with doramectin, 1 mL/50 Kg b.w.); G3 and G4: animals treated with 2.5 and 5 mg/Kg of CP extract, respectively.

The *in vivo* trials on the anthelmintic activity of CP extract showed significant reduction in the mean faecal egg count throughout experimental period (Figure 1(a)). The egg excretion pattern was similar for the positive control (G2) and treated groups (G3 and G4). However, a significant difference (*P* < 0.05) was observed between the negative control (G1), G3 and G4, which received the extract doses of 2.5 and 5.0 mg/Kg b.w., respectively. All groups treated with this extract had a positive FEC reduction of 54.61% for G3 (2.5 mg/Kg b.w.) and 71.21% for G4 (5.0 mg/Kg b.w.). Regarding the change in live body weight, no statistically significant difference was observed among G1, G2, and G3. However, the G4 gained a significant difference in body weight (*P* < 0.001) (Figure 1(b)).

### 3.2. Haematological Profiles

PCV values did no change during experimental period. The average values were not significantly different between the extract and Doromectin treated groups (G2, G3, G4) and the untreated group (G1). For leukocyte counts, similar results were observed in the posttreatment groups and also no significant differences were seen in the eosinophil values in blood (*P* > 0.05) when compared to the negative and positive control. Basically, the counts remained stable throughout the experiment without any difference for all groups.

### 3.3. Serum Antibody Responses

**Figure 2:**
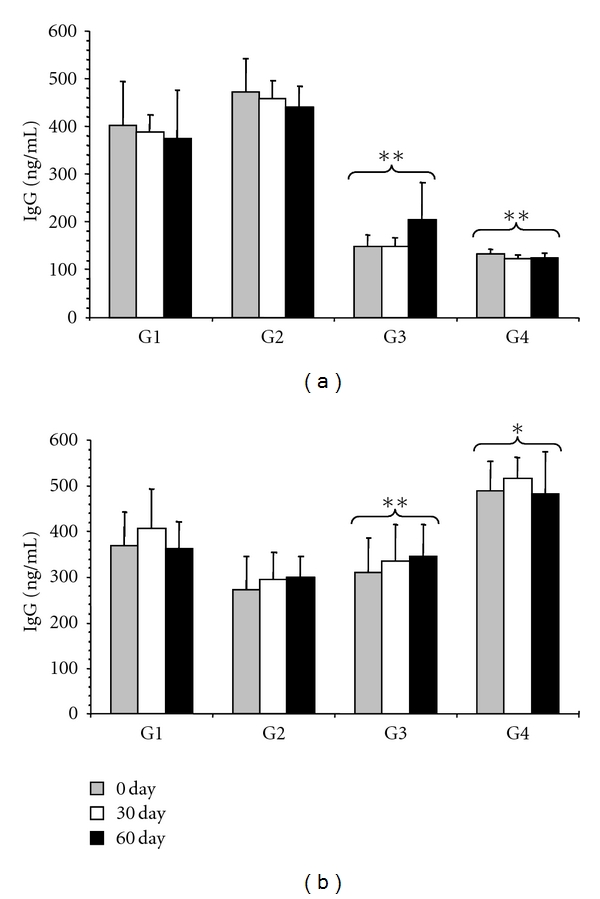
Mean concentrations of serum IgG (a) and IgA (b) in goats naturally infected with gastrointestinal nematodes, traited with 2.5 and 5 mg/Kg b.w of CP extract. Values are expressed as means ± standard deviation. Significant differences are indicated by an asterisk marks; (*P < 0.05; **P < 0.0001). G1: negative control (untreated); G2: positive control (treated with doramectin, 1 mL/50 Kg b.w.); G3 and G4: animals treated with 2.5 and 5 mg/Kg of CP extract, respectively.

**Figure 3:**
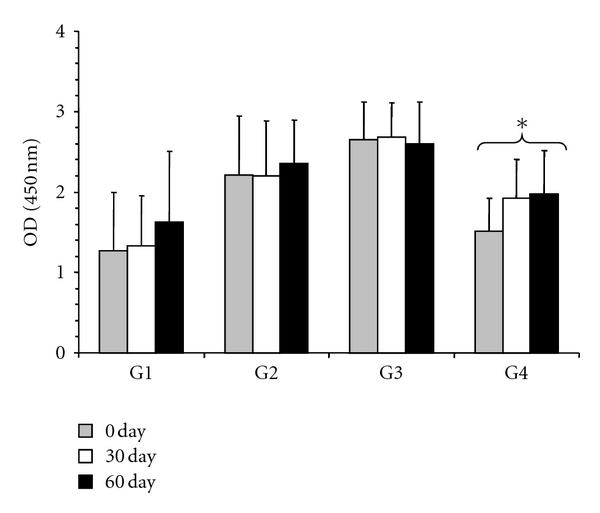
Optical density (OD) variations in ELISA Serum-specific IgG response using adult *H. contortus* antigenic extract in naturally infected goats, treated with doramectin and CP extract. Optical densities were measured by ELISA at 450 nm. Significant differences are indicated by an asterisk marks (*P* < 0.05). G1: negative control (untreated); G2: positive control (treated with doramectin, 1 mL/50 Kg b.w.); G3 and G4: animals treated with 2.5 and 5 mg/Kg of CP extract, respectively.

The average values of serum concentration for IgG and IgA are presented in Figure 2. In the positive control (G2), a significant rise in serum IgG (*P* < 0.001) was observed, whereas in the CP extract-treated groups (G3 and G4), IgG levels were low, with a significant decrease (*P* < 0.001) as shown in Figure 2(a). In addition, serum IgA levels had a significant response (*P* < 0.05) in the 5.0 mg CP extract-treated group (G4; Figure 2(b)). The highest concentrations of IgA were detected in samples from G3 and G4. The concentration of G4 was 488.69 ng/mL at the baseline moment, 517.12 ng/mL at 30 days, and 483.51 ng/mL 60 days later. With the exception of the variation observed for G3, which showed the highest IgA concentration after 60 days of treatment, being 309.04 ng/mL before treatment, 336.18 ng/mL at 30 days, and 345.72 ng/mL at 60 days after treatment, the remaining groups had an increasing concentration values from baseline until 30 days and decreasing concentration at 60 days. Moreover, the serum anti-*Haemonchus* antibody responses presented significant increases in G2 and G3, whereas G4 yielded an average OD of 1.52 ± 0.40, 1.93 ± 0.48, and 1.98 ± 0.55 for the three collected sample periods, presenting a decline when compared with those obtained in G3 (Figure 3).

## 4. Discussion

This work was carried out in order to find a phytotherapic to help control GIN in small ruminants. The parasitological analysis indicated that the CP extract exhibited anthelmintic activity in naturally infected goats, whose effectiveness was evidenced by the reduction in FEC as compared to the negative control group. Probably the higher deviations on the determined FEC values were due to the traditional and extensive management practices in the Brazilian semiarid region; therefore, the goats were always exposed to a contaminated environment. In addition, should be considered the differences between animals and their susceptibility to nematode infection [15, 16]. A similar study with a hydroalcoholic extract of *C. crista*, a plant of the same genus, showed *in vivo* a significant FEC reduction [5].

Under the experimental conditions, the results with *C. pyramidalis* extract also provided evidence of a reduction in nematodes of goats compared to controls. A possible explanation of this reduction may be due to the direct effect of CP extract on parasite leading to drop in FEC in natural infected goats. One of the effects resulting from the extract treatment could be due to the decrease of fecundity of female parasites, which can be explained by the presence of active compounds in this plant extracts.

The active principles for the anthelmintic effect of *C. pyramidalis* have not been exactly identified so far. However, its phytochemical analysis revealed the presence of different chemical compounds, among them polyphenols, like tannins and flavonoids [11, 12], that could be considered as the responsible for this bioactivity [16, 17]. Once some plants have been reported to have anthelmintic properties, attributed to secondary metabolites (e.g., saponins, lignans, tannins, and other polyphenolic compounds), some of them were associated with antiparasitic effect [4]. Thus, the observed FEC reduction in this work is consistent with other studies that have reported that plant extracts rich in polyphenolic compounds induced anthelmintic activity, involving experimental animals that have been used in trials to validate medicinal activities [9, 10, 13, 18].

Despite a significant decline in mean FEC, blood parameters did not change significantly throughout the experiment between any groups. Similarly, the extract does not appear to have influenced the change of weight in animal groups during the experimental period. However, the G4 animals treated with 5.0 mg/Kg b.w. were the only ones to present a significant weight gain. This concentration appears to be the most efficient.

All these parameters are considered to be important element in the response against GIN infection. Although controversial, this is consistent with other experimental findings with helminthes, in which no notable alteration in these parameters was found [19].

In the present experiment, it is likely that another mechanism of the CP extract affects against GIN. The mechanisms whereby the consumption of certain plants and plant extracts can affect parasite cycle both *in vitro* and *in vivo* are unknown. However, their consumption can be associated with an enhanced immune response of the host towards the parasites [20].

In the study of humoral immune response, antibodies of two immunoglobulin classes were investigated: IgA and IgG. A significant increase in IgA levels was observed in G4, while low levels of IgG were observed in groups G3 and G4, corresponding to the animals treated with the CP extract. From the present results and considering the significant reduction in the egg output, this study indicates that IgA may play a role in the parasite control. It appears that the IgA response is involved with the generation of protective immunity against GIN, as parasitological data remained low in animals from G3 and G4 throughout the experiment. Although this was a significant response, no relationship was found between IgA and FEC.

Previous studies in small ruminants suggest that IgA may be the major immunological mechanism of either reduced fecundity and/or eliminate adult worms [21–24]. IgA levels could reflect the degree of sensitisations of the animal. Therefore, serum IgA might indicate a high level of resistance to reinfection against GIN [25].

The present results also support that *C. pyramidalis*, which showed the presence of phenolic compounds in phytochemical analysis and is involved in anthelmintic activy, as demonstrated by several experiments [13, 17], provoked a protective immune response when the animals were treated with this extract [26]. Phenolic compounds were also evaluated *in vitro* for their antimicrobial activity to control pathogenic bacteria [4, 17, 27].

The role of these substances needs to be better established because plants with anthelmintic properties can be one key factor to the control of nematode infections, whereas a number of medicinal plants have been used to treat parasitic infections in man and animals [4, 17]. According to Siqueira et al. [10], *C. pyramidalis*, a plant with antimicrobial indications, showed a higher content of tannins. Our experimental results suggest a possible relationship between these compounds and the observed activity in goats.

Therefore, further experiments with infected animals are essential to evaluate the best dose of the extract to be administered and still offer subsidies for the development of new drugs for the treatment of livestock against gastrointestinal nematodes.
